# Ultrashort Echo Time Magnetic Resonance Morphology of Discovertebral Junction in Chronic Low Back Pain Subjects

**DOI:** 10.3390/tomography11020012

**Published:** 2025-01-23

**Authors:** Palanan Siriwananrangsun, Tim Finkenstaedt, Karen C. Chen, Won C. Bae

**Affiliations:** 1 Department of Radiology, Faculty of Medicine Siriraj Hospital, Mahidol University, Bangkok 10700, Thailand; palanan.siri@gmail.com; 2 Department of Radiology, University of California-San Diego, San Diego, CA 921093, USA; 3 Institute of Diagnostic and Interventional Radiology, University Hospital of Zurich, University of Zurich, 8006 Zurich, Switzerland; tim@finkenstaedt.ch; 4 Department of Radiology, VA San Diego Healthcare System, San Diego, CA 92161, USA; karenchanchen@gmail.com

**Keywords:** low back pain, lumbar spine, cartilage endplate, intervertebral disc, disc degeneration, ultrashort echo time

## Abstract

*Background:* Chronic low back pain (LBP) has been associated with intervertebral disc (IVD) degeneration, but its association with abnormal morphology at the discovertebral junction (DVJ) is unclear. The goal of this study was to evaluate the DVJ morphology in asymptomatic (Asx) and symptomatic (Sx) subjects for LBP using ultrashort echo time (UTE) MRI. *Methods:* We recruited 42 subjects (12 Asx and 32 Sx). Lumbar IVD degeneration was assessed using Pfirrmann grading (1 to 5), while the abnormality of DVJ (0 = normal; 1 = focal; 2 = broad abnormality) was assessed using UTE MRI. The effects of LBP and level on the mean IVD and DVJ grades, the correlation between IVD and DVJ grade, and the effect of LBP and age on the number of abnormal DVJs within a subject were determined. *Results:* IVD grade was higher in Sx subjects (*p* = 0.013), varying with disc level (*p* = 0.033), adjusted for age (*p* < 0.01). Similarly, DVJ grade was also significantly higher in Sx subjects (*p* = 0.001), but it did not vary with DVJ level (*p* = 0.7), adjusted for age (*p* = 0.5). There was a weak positive (rho = 0.344; *p* < 0.001) correlation between DVJ and IVD grade. The total number of abnormal DVJs within a subject was higher in Sx subjects (*p* < 0.001), but not with respect to age (*p* = 0.6) due to a large spread throughout the age range. *Conclusions:* These results demonstrate the feasibility of using in vivo UTE MRI of the lumbar spine to evaluate the DVJ and the correlation of DVJ with LBP. This study highlights the need for a better understanding of DVJ pathology and the inclusion of DVJ assessment in routine lumbar MRI.

## 1. Introduction

Chronic low back pain (LBP) is a significant health concern with a high socioeconomic cost [1]. Affecting millions worldwide, LBP not only diminishes the quality of life for those afflicted but also imposes a substantial economic burden due to healthcare costs, lost productivity, and disability. The pervasive nature of LBP underscores the need for a deeper understanding of its underlying causes and effective diagnostic and therapeutic strategies.

While the exact etiology is unclear, LBP has been associated with intervertebral disc (IVD) degeneration [2] and vertebral endplate lesions such as Schmorl’s nodes [3]. IVD consists of distinct regions, including the nucleus pulposus (NP), annulus fibrosus (AF), and superior and inferior cartilaginous endplates (CEP), which are attached to the bony vertebral endplates [4] at the discovertebral junction (DVJ). The CEP is a 1 to 2 mm thick tissue [5] that not only plays a role in the mechanical stability of the spine but also as a pathway for molecular transport [6]. While studies have focused on the natural history of IVD degeneration [2,7], studies on the degeneration of the tissues at the DVJ are sparse.

A large body of work on lumbar spine degeneration has been performed using magnetic resonance imaging (MRI) as a routine evaluation tool [8,9,10,11,12,13,14,15,16]. MRI protocols, including sagittal spin echo T2-weighted [17] sequence, are used for the Pfirrmann grading [16] of IVD degeneration, assessing IVD structure, signal intensity, and height to determine five grades from 1 (normal) to 5 (complete degeneration and collapse of joint space).

In contrast to IVD, the DVJ has been difficult to image using routine MRI due to the short T2 properties of the CEP [18,19,20], whose signal decays quickly and becomes indistinguishable from the adjacent bony endplate. Ultrashort echo time (UTE) MR imaging overcomes this limitation, acquiring a signal from short T2 tissues [21,22,23,24,25,26,27,28,29,30]. Using UTE source images from different echo times and digital subtraction to enhance the contrast of short T2 components [25], an MR signal from the CEP can be acquired with high signal intensity [18,20,31,32], making it distinct from the adjacent tissues. In human lumbar spines, the normal and the predominant UTE MR morphology of the DVJ in the sagittal plane is that of a continuous, linear, and high signal intensity [18,20,32] from the CEP, consistent with the anatomy [4,18]. Abnormalities in the CEP morphology may include the loss of the signal and irregular appearances [18,20,32].

In a recent study performed in cadaveric human spines [20], about a third of all CEP evaluated were abnormal, and a significant association between degeneration of the CEP and the IVD was found. A major limitation of this study was the use of cadaveric samples instead of live subjects, as well as the fact that the LBP status in the donors was not known.

The goal of this study was to evaluate the DVJ in asymptomatic and LBP subjects, to compare the prevalence of abnormal UTE MR morphology, and to compare it with IVD degeneration.

## 2. Materials and Methods

### 2.1. Study Design

This is a prospective cross-sectional study designed to compare the MRI findings of IVD degeneration and DVJ abnormality between subjects with and without chronic low back pain.

### 2.2. Subjects

A total of 42 subjects were recruited. These included 12 asymptomatic (Asx) volunteers (mean ± standard deviation of age = 37.1 ± 10.4 years old; 6 males, 6 females) and 32 symptomatic (Sx) subjects (49.0 ± 16.8 years old; 22 males, 12 females). The inclusion criterion for the symptomatic subjects was persistent low back pain for 4 to 6 weeks prior to MRI. Exclusion criteria for all subjects included age < 19 years old and any condition that would be contraindicated for MRI, such as non-MRI-compatible implants, metallic foreign body, pregnancy, high body weight (>300 lbs), and claustrophobia. Table 1 summarizes the number of subjects and age range by group.

### 2.3. MRI

**Figure 1:**
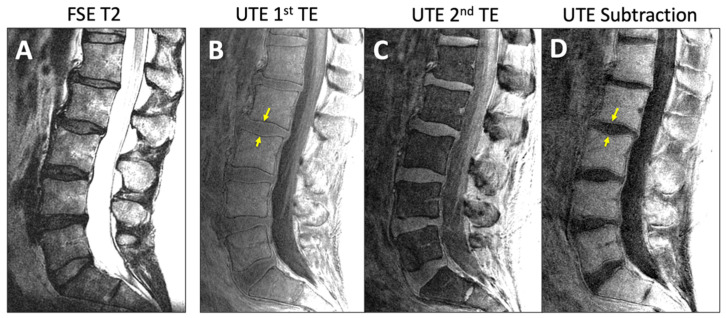
Representative sagittal MR images of lumbar spine of an asymptomatic subject acquired with (**A**) conventional fast spin echo T2 (FSE T2) and (**B**–**D**) ultrashort echo time (UTE) sequences. UTE MR images from (**B**) 1st TE minus (**C**) 2nd TE yielded (**D**) the subtraction image. Yellow arrows indicate the DVJ.

Imaging was performed on a clinical 3-Tesla system (General Electric Discovery 750) with a posterior spine coil. The imaging protocol included (1) sagittal fast spin echo (FSE) T2-weighted (Figure 1A) with scan parameters of repetition time (TR) = 4600 milliseconds (ms); echo time (TE) = 102 ms; acquisition matrix = 224 × 224; slice thickness = 3 mm (mm); field of view (FOV) = 240 mm; and scan time = 2:14 min; and (2) 3D UTE (Figure 1B,C; TR = 40 ms; TE = 0.03 and 4.6 ms; number of spokes = 6000; matrix = 256 × 256; slice = 2 mm; FOV = 24 cm; flip angle = 2 degrees; and scan time = 4:00 min.

**Figure 2:**
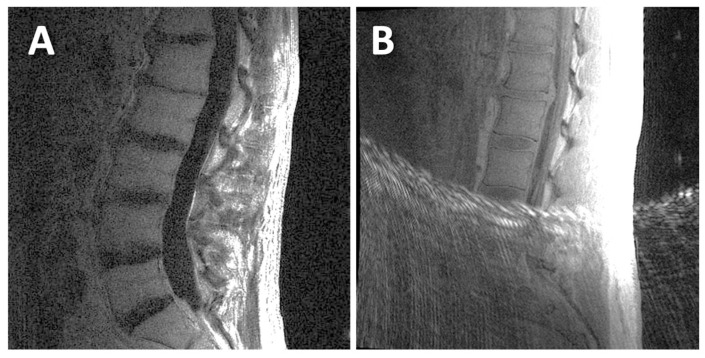
Examples of UTE MR images that were difficult to evaluate: (**A**) this UTE subtraction image had a low signal-to-noise ratio; (**B**) this UTE 1st TE image had streak artifacts that obstructed the inferior half of the image.

For UTE, both the 1st TE image (Figure 1B) and the digital subtraction image (1st TE minus 2nd TE; Figure 1D) were evaluated for DVJ morphology. While the subtraction image generally provided good contrast and detail for the DVJ, the 1st TE image was evaluated alongside the subtraction image if the subtraction image was too noisy (e.g., Figure 2A) (roughly a third of all cases). Additionally, when a UTE image contained streak artifact (Figure 2B) (occurring in 4 cases), only the unobstructed portion of the image was evaluated.

### 2.4. MRI Evaluation

**Figure 3:**
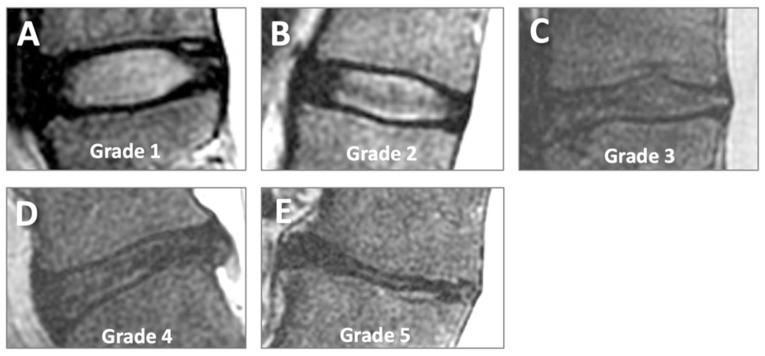
Pfirrmann grading of disc degeneration on sagittal conventional T2-weighted MR images. Grades 1 (**A**), 2 (**B**), 3 (**C**), 4 (**D**), and 5 (**E**) represent increasing severity of disc degeneration.

MRI evaluation was performed on the mid-sagittal image showing the largest IVD cross-section. On FSE T2 images, the Pfirrmann grading scheme [16] was used to evaluate IVD degeneration in an ordinal scale from grade 1 (normal) to grade 5 (complete degeneration and collapse of joint space), as shown on Figure 3. We considered the disc structure, signal intensity, and disc height. All fully visible IVDs from T12/L1 to L5/S1 (6 discs) were graded (a total of 144 discs).

**Figure 4:**
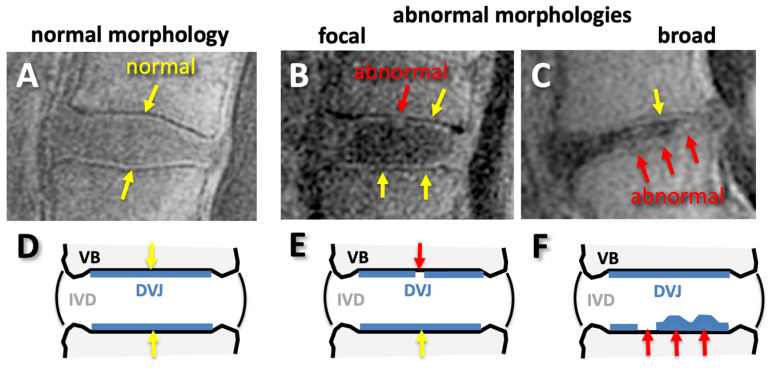
Normal (**A**,**D**) and abnormal (**B**,**C**,**E**,**F**) morphology of discovertebral junction (DVJ) seen on sagittal UTE MR images (**A**–**C**) and on illustrations (**D**–**F**). The normal morphology was continuous, distinct, linear, and high signal intensity (**A**,**D**), while any deviations from normal, such as signal loss or irregular thicknesses found focally (**B**,**E**) or broadly (**C**,**F**), were considered abnormal.

For DVJ morphology, each DVJ was evaluated ordinally as follows: a normal DVJ morphology was considered a distinct and continuous line of high signal intensity (Figure 4A) and was given a DVJ grade of 0, while an abnormal DVJ morphology was any deviation from this, including obvious signal loss, thickening, or irregularity [32]. Here, a focal abnormality (roughly < 5 mm wide) was graded as 1 (Figure 4B), while a broad-based abnormality (roughly > 5 mm wide) was graded as 2 (Figure 4C). While this was not an objective criteria, the focal abnormalities were often narrow and distinct, while broad-based abnormalities were not distinct (i.e., Figure 4C). DVJs at spinal levels of L1 superior to S1 superior were graded (total of 321 DVJs).

### 2.5. Statistics

We initially performed a *t*-test [33] and found that the Sx group had a significantly higher (*p* = 0.04) mean age. Therefore, we adjusted for age in the following analyses whenever possible. The proportion of the sexes between the Asx and Sx groups was not different (chi-square *p* = 0.4), so sex was not considered in the remaining analyses.

(1) To determine the effect of low back pain and disc level (L1/2, L2/3, L3/4, L4/5) on the mean IVD grade with age as a covariate, repeated measures analysis of variance (rmANOVA) [34] was used. Similar analysis was performed to determine the effect of low back pain and DVJ level (L1 superior to L5 superior; 8 levels) on the mean DVJ grade with age as a covariate. While the IVD and DVJ grades are ordinal variables, as opposed to continuous variable, this model was deemed reasonable since the residuals were symmetrically distributed. While sample sizes were limited by the availability of suitable subjects, with a combined total of 44 subjects, we had a moderate power (0.74) to distinguish DVJ grade between the Asx and Sx subjects, with an effect size of ~0.3 and a significance level set at 0.05.

(2) To determine if the number of abnormal DVJs (grades 1 or 2, regardless of the level) in a subject increases with aging in the low back pain group, we also performed analysis of covariance (ANOVA) [35,36], with low back pain as the fixed factor and age as the covariate.

(3) To determine the differences between the proportions of IVD or DVJ grades between the Asx and Sx groups, a contingency table with the chi-square test [37] was used.

(4) Finally, to determine if IVD grade correlates with DVJ grade, Spearman’s rank correlation [38] was used.

For all tests, the significance level was set at 5%. Statistical analyses were performed using Systat software (v12, Grafiti LLC, Palo Alto, CA, USA) or JASP software (version 0.18.3, jasp-stats.org).

## 3. Results

### 3.1. MRI Appearances

**Figure 5:**
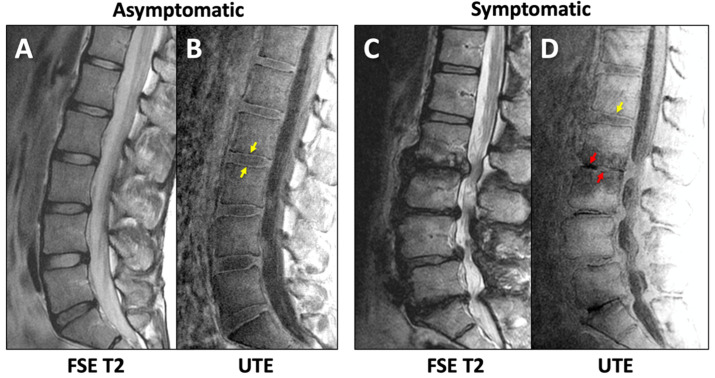
Sagittal lumbar imaging of asymptomatic (**A**,**B**) and symptomatic (**C**,**D**) subjects using FSE T2 (**A**,**C**) and UTE (**B**,**D**) techniques. Normal (yellow arrows in (**B**,**D**)) and abnormal (red arrows in (**D**)) morphology of the DVJ can be seen in the UTE MR images.

Unlike the FSE T2 images that depicted DVJ with low signal intensity (Figure 5A,C), UTE MR images showed distinct lines of high signal intensity in the majority of the DVJs in the Asx subjects (Figure 5B,D, yellow arrows). In contrast, UTE MR images of many of the Sx subjects showed DVJs that appeared irregular or lost signal intensity (Figure 5D, red arrows). In the Sx subjects, we found morphologic degenerative endplate changes in the vertebral body (i.e., Modic change). While conventional FSE T2-weighted sequences (Figure 5C) were able to differentiate between the marrow edema and fatty endplate degeneration subjacent to the vertebral endplate, UTE images (Figure 5D) were able to directly visualize changes in the cartilaginous endplate.

### 3.2. IVD Degeneration with LBP, Level, and Age

**Figure 6:**
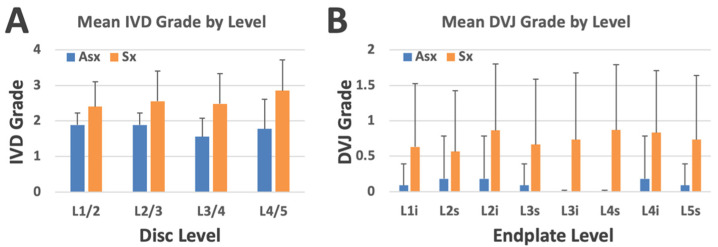
(**A**) Mean IVD grade by disc level and low back pain group. (**B**) Mean DVJ grade by DVJ level (where “i” and “s” refer to inferior and superior sides, respectively) and low back pain group. Bars represent standard deviation.

IVD grade varied significantly with LBP (*p* = 0.013) and disc level (*p* = 0.033), adjusted for age (*p* < 0.01). Figure 6A shows the distribution of mean disc grades across different disc levels and LBP groups. The mean disc grade for the Sx group was greater than 2 at every level, with the highest value at L4/5. In contrast, the mean disc grade for the Asx group was less than 2, with the highest values at L1/2 and L2/3.

The proportions of the IVD grades between the Asx and Sx groups assessed with the contingency table (Table 2) were significantly different (Chi-square = 28.5, *p* < 0.00001), where the majority of the Asx subjects had IVD grade 2 and below, while the Sx subjects had IVD grade 2 and higher.

### 3.3. DVJ Abnormality with LBP, Level, and Age

DVJ grade varied significantly with LBP (*p* = 0.001) but not with DVJ level (*p* = 0.7), adjusted for age (*p* = 0.5). Figure 6B shows the distribution of mean DVJ grades across different disc levels and LBP groups. The mean DVJ grade for the Sx group was greater than 0.5 at every level, with the highest value at L4 superior. In contrast, the mean DVJ grade for the Asx group was consistently less than 0.2, showing a marked difference.

The proportions of DVJ grades with respect to the Asx and Sx groups assessed with the contingency table (Table 3) were significantly different (chi-square = 36.5, *p* < 0.00001), where the majority of Asx subjects had DVJ grade 0 (normal), while the Sx subjects had much greater percentages of DVJ grades 1 and 2 (both are abnormal).

**Figure 7:**
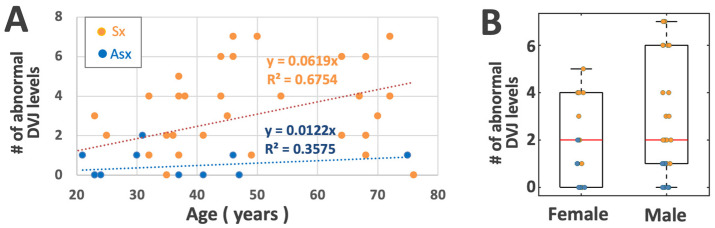
(**A**) Scatter plot of the number of DVJ levels within a person with an abnormal DVJ (grades 1 or 2) vs. age, stratified by low back pain group. (**B**) Box plot stratified by sex and low back pain group.

When the number of abnormal DVJs (grades 1 or 2, regardless of the level) in a single subject was plotted against the age (Figure 7A), using ANCOVA, we found a significant effect of LBP (*p* < 0.001), consistent with relatively greater numbers in Sx compared to Asx subjects, but an insignificant effect of age (*p* = 0.6), consistent with the large spread of the data throughout the age range. Anecdotally, however, there appeared to be an increase in the upper limit of this number with respect to ages ranging from roughly 20 to 50 years old, staying flat thereafter. A similar box plot, stratified by sex and low back pain (Figure 7B), suggested that the median number of abnormal DVJs was similar with respect to both sexes, but males tended to have a broader spread.

### 3.4. Correlation Between IVD and DVJ Grades

A weak positive (Spearman’s rho = 0.344) significant (*p* < 0.001) correlation was found.

## 4. Discussion

The results of our investigation underscore the potential of UTE MRI in the evaluation of the DVJ and its associated pathologies within the lumbar spine. Our study demonstrates that although improvements are needed, acquiring UTE MR images of the lumbar spine of diagnostic quality is feasible in human subjects. We found a significant effect of chronic low back pain on the presence of DVJ abnormalities in tandem with IVD degeneration.

The number of studies on the DVJ or CEP using UTE MRI is increasing but still relatively small. Our early study established a histoanatomical basis for CEP as the tissue structure giving the linear signal intensity seen on the MRI [18]. Subsequent studies applied UTE MRI in human subjects [39,40], finding associations between DVJ abnormality and IVD degeneration [20,39,40]. While these studies were performed in patients with low back pain, a comparison against asymptomatic subjects was not performed. The present study is intended to fill this gap in our knowledge.

Our findings of greater IVD degeneration with low back pain and the variations with disc level and age are in close agreement with past studies. IVD degeneration is highly prevalent among those with LBP, accounting for up to 42% of patients [41]. A meta-analysis of 280 MR studies found that degenerative IVD pathologies are significantly more prevalent in adults with LBP compared to Asx subjects [42]. Level-wise trends were also consistent; our study found the highest IVD grades at the lower levels of L4/5 and L5/S1 among LBP patients, corroborating past studies [7,43].

Our discovery that DVJ grade increases significantly with LBP is a new but an inferable finding. Our recent work on cadaveric spines [20], as well as other in vivo studies [39,40], found a significant association between the presence of abnormal DVJ and IVD degeneration. Since IVD degeneration is more prevalent in LBP subjects, DVJ abnormality would be expected to be more prevalent as well. Nonetheless, to our knowledge, this is the first direct comparison of the mean DVJ grade and the proportions of the DVJ grades between Asx and Sx subjects. The correlation between the number of abnormal DVJs in each subject vs. age (Figure 7) suggested that Sx subjects were more likely to have abnormal DVJ, regardless of the age.

Which alterations result in DVJ abnormality on UTE MRI and how this may cause LBP is still unclear. The experimental removal of CEP resulted in the loss of the normal DVJ appearance, i.e., linear high signal intensity [18]. In vivo, however, more likely events may include focal or broad build-up of calcification in the CEP [44,45], which can result in a loss of signal intensity [46], as well as physical disruption of the DVJ, alongside the disruption of the adjacent bony vertebral endplate (i.e., Schmorl’s node formation) [32]. Since vertebral endplate is highly innervated, and the density significantly increases with vertebral endplate pathology [47], this may be one reason for a strong association of abnormal DVJ with LBP found in our study. Speculatively, if abnormal DVJ causes IVD degeneration [20], this would be an indirect mechanism of LBP as well.

There are several limitations to this study. The number of subjects was modest, but more Sx subjects than Asx subjects were included. The difference in age between the Asx and Sx groups is also an issue, as we have previously found a trend of an increasing prevalence of abnormal DVJ with advancing age [20]. For this reason, we adjusted for age in our statistical analyses wherever possible. Another limitation is the lack of body weight information; given that individuals with high body weight are generally more susceptible to low back pain, it may be important to isolate the confounding effects of body weight and low back pain when it comes to DVJ abnormality. The cross-sectional nature of this study limits the interpretation, as the incidence and progression cannot be determined. This study had only a single reader; however, we have established a substantial inter-reader agreement for UTE MR assessment of the DVJ [32]. For this study, we arbitrarily classified abnormal DVJ into two categories (focal and broad), but more granular scoring may also be used [40]. Lastly, the use of quantitative UTE MRI to quantify the T2* of the CEP is also actively being investigated [31,48,49,50], but this study focused on the morphologic findings as an initial step.

## 5. Conclusions

In conclusion, our study highlights the feasibility of incorporating UTE MRI for the evaluation of DVJ, as well as a strong association of DVJ abnormality with LBP. A comprehensive understanding of the DVJ with respect to what causes these abnormal morphologies and why they associate with LBP will be helpful in the development of diagnostic criteria and therapeutic approaches. Future work may include longitudinal studies to determine the sequence of events related to DVJ abnormality and IVD degeneration in healthy and LBP subjects.

## Figures and Tables

**Table 1:** Number (#) of subjects and their age range by low back pain group.

Group	Total #	Age Range	Male #	Age Range	Female #	Age Range
Asx	12	21 to 75	6	23 to 75	6	21 to 37
Sx	32	19 to 76	0	32 to 76	0	19 to 75
Total	44	19 to 76	26	23 to 76	18	19 to 75

**Table 2:** Prevalence of IVD Pfirrmann grades (grades 1 (or G1) to grade 5 (or G5)) in low back pain groups of asymptomatic (Asx) and symptomatic (Sx) participants.

**Disc**	**G1**	**G2**	**G3**	**G4**	**G5**
**Asx**	27.8%	66.7%	5.6%	0%	0%
**Sx**	4.6%	48.1%	34.3%	11.1%	1.9%

**Table 3:** Prevalence of DVJ grades (G0 = normal; G1 = focal abnormality; and G2 = broad abnormality) in low back pain groups of asymptomatic (Asx) and symptomatic (Sx) participants.

DVJ	G0	G1	G2
**Asx**	93.8%	2.5%	3.8%
**Sx**	56.8%	12.4%	30.7%

## Data Availability

The data that support the findings of this study are not publicly available due to reasons of sensitivity. Anonymized data may be made available from the corresponding author upon review of the request. Data are located in controlled access data storage at the corresponding author’s institution.
